# Exploring the Utility of Digital Voice Assistants for Primary Care Patients, Including Those With Physical and Visual Disabilities: Cross-Sectional Study

**DOI:** 10.2196/66185

**Published:** 2025-08-14

**Authors:** Maya Rajan, Allison Furgal, Reema Kadri, Omar Arman, Kate Panzer, Donna Wicker, Michael M McKee, Melissa Plegue, Alexandria Degner, Lorraine R Buis

**Affiliations:** 1 Department of Family Medicine, University of Michigan, 1018 Fuller Street, Ann Arbor, MI, 48104, United States, 1 734-998-7124; 2 Department of Biostatistics, University of Michigan, Ann Arbor, MI, United States; 3 Department of Orthopedic Surgery, University of Virginia, Charlottesville, VA, United States; 4 Department of Ophthalmology and Visual Sciences, University of Michigan, Ann Arbor, MI, United States; 5 Detroit Mercy Eye Institute, University of Detroit Mercy, Novi, MI, United States; 6 Department of Pediatrics, University of Michigan, Ann Arbor, MI, United States

**Keywords:** smartphone, mobile applications, disabled persons, visually impaired persons, primary health care, digital voice assistant

## Abstract

**Background:**

Today, most smartphones provide a digital voice assistant (DVA) for their user, and it is estimated that about 91% of adults report owning and operating a smartphone. A DVA is an automated system preinstalled on technological devices, such as smartphones, computers, tablets, and speakers, which serves to aid users in performing tasks like answering questions, managing smart devices at home, playing music, managing schedules, sending messages, and more. Research with DVA is emerging, and its applicability to health and health care needs to be elucidated.

**Objective:**

The objective of this study was to describe the use of DVAs among primary care patients, as well as purposely sampled clinics including patients with visual and physical disabilities.

**Methods:**

A convenience sample of adult participants was recruited to complete a needs assessment survey to ascertain the interest and possible utility of DVAs to promote and enhance health from among three populations at an academic medical center: (1) general primary care patients recruited from a primary care clinic, (2) patients with visual disabilities recruited from a low vision clinic, and (3) patients with physical disabilities recruited from a physical medicine and rehabilitation clinic. The survey used in this study was a 46-item investigator-developed instrument administered to participants assessing knowledge, use, and perceptions of DVAs, participant interest to participate in related future studies, and demographics.

**Results:**

The results of the survey showed that the majority of participants have used a DVA before (69.7%, 152/218) and were or might be willing to use them in the future (84.0%, 178/212). Participants reported moderate to high concern about the privacy (47.8%, 97/203), security (54.5%, 110/202), and confidentiality (51.7%, 105/203) of DVAs. A greater proportion of those with visual disabilities reported having never used DVA than those without visual disabilities (39.0% vs 24.6%, *P*=.03). There was no significant difference in reliance on DVAs for participants with and those without physical disabilities (45.0% vs 34.9%, *P*=.31), indicating that they do not require it for everyday needs.

**Conclusions:**

DVA use remains low among the surveyed participants with physical and visual disabilities. For those with visual disabilities, DVA use was seen to be advantageous in everyday life for tasks such as answering questions and seeking information, but not for those with physical disabilities. However, further research should be conducted that focuses on the use of DVAs by accessing data that represent an individual’s DVA use without being biased by knowledge of a research study. In addition, research is needed on DVA use that includes diverse samples of participants with physical and visual disabilities, which address the barriers to using DVAs for these adult populations.

## Introduction

Currently, about 91% of American adults report owning and operating a smartphone [1] and the overwhelming majority of those smartphones provide users with a digital voice assistant (DVA). DVAs are software that comes preinstalled on many different mobile devices, such as smartphones, computers, tablets, and laptops, or that operates as a stand-alone device such as a smart speaker. DVAs aid users in performing tasks like answering questions, managing smart devices in the home, playing music, managing schedules, sending messages, and more. To recognize and execute these tasks, DVAs communicate with the user in their natural language by using voice recognition while using either native or third-party services to obtain the requested information or perform the task at hand. The first DVA, Siri, was invented in 2011 by Apple and additional companies brought their own DVAs to market in the following years. DVAs not only exist on smartphones, but also within other smart devices such as speakers, TVs, and other various automated home control systems [2]. Studies investigating the use and quality of these smart home devices have shown that they increase convenience, reduce loneliness, and improve quality of life in older adults and adults with disabilities [3]. However, barriers exist in installing, troubleshooting, and updating these technologies for long term use [4]. The most prevalent examples of DVAs in the United States across smartphones and home devices include Siri by Apple, Alexa by Amazon, and Google Assistant [5]. DVAs have experienced explosive growth within the past few years, with recent estimates indicating 62% of American adults having used one on a device [6].

In the past, most research involving DVAs has been narrowly focused and limited to economic analyses focused on potential market value [5], as well as consumer perceptions of privacy and security in regards to DVAs [7]. DVA applications within health care are becoming more widely commercially available and have been primarily focused on demonstrative projects for memory support in patients with dementia [8], older adults with diabetes [9], and communicative apps and technologies for individuals with intellectual and developmental disability or autism spectrum disorder [10]. DVAs are ripe for study within health care contexts, and they pose a particularly exciting innovation in the realm of assistive technologies for older populations, as well as those with various disabilities. Indeed, the potential for DVAs to aid older adults is becoming more clear and emerging research, especially during the COVID-19 pandemic, has investigated the ability of DVAs to reduce loneliness and social isolation of older adults [11].

Aside from assisting with disabilities, older populations engage with DVAs in more relational ways, such as through asking the DVA “personal questions” to get to know them, asking for advice, and engaging with the DVA to alleviate stress [12]. Indeed, as DVAs are typically controlled with voice activation and voice prompts, they may be more easily used by populations who have physical or visual disabilities that result in difficulties manipulating or navigating their mobile devices. DVAs have the potential to deliver interventions specifically for patient populations with a broad range of physical and visual disabilities, both of which can make using mobile devices challenging. For individuals whose physical disabilities affect their endurance, mobility, or dexterity, DVAs could enhance their ability to complete daily tasks and acquire information by mitigating detrimental exertion. Likewise, those with visual disabilities may benefit from DVAs ability to read information out loud instead of being required to visually read online information.

DVAs have great potential for use in health care and could potentially facilitate clearer communication between patients and providers through language translation capabilities, hands-free calling and messaging, and reminders for medications and rehabilitation exercises [13]. For those with visual disabilities, as well as physical disabilities that prevent one from holding a device, DVAs could be transformative in bridging their connection to online health information, family members, and health care providers [14]. The technology can provide greater independence and manage safety risks for those who fall under these specific populations [15]. Given the widespread penetration of DVAs, coupled with their potential for use among populations who may struggle with traditional mobile devices, research to explore potential applications for use within these contexts is needed. In addition, recent and ongoing research exists to evaluate the accuracy and quality of health information that DVAs provide to users [16]. As a first step at understanding the potential for greater use within health care settings, it is integral to investigate the behaviors and opinions on DVAs and barriers to access and use of this technology.

To better understand the potential for use in various health care settings and populations, we sought to describe general DVA use among a broad range of adult patients including primary care patients, as well as those seeking care from specialty clinics where there are high concentrations of patients with either visual or physical disabilities. The results of this study carry importance to inform the future innovations in DVA technology. Depending on the use type, or lack thereof, these tools can be better designed to address the needs of these populations and aid them in their daily functioning.

## Methods

### Participants and Setting

A cross-sectional study using a convenience sample of adult participants was used to survey the use and perceptions of DVAs from patients at Michigan Medicine, the academic medical center affiliated with the University of Michigan. Patients from 3 purposely sampled clinics in a single city were recruited. The clinics included a family medicine (Briarwood Family Medicine Center), low vision (Kellogg Eye Center Low Vision and Rehabilitation Clinic), and physical medicine & rehabilitation (PM&R) clinic at Michigan Medicine. The family medicine clinic was selected to reflect a typical primary care setting with a range of patients. The low vision clinic was selected for its access to patients with visual disabilities. Finally, the PM&R clinic was selected to enrich the sample for individuals who had physical-related disabilities. Potential participants younger than 18 years were excluded from participation.

### Participant Recruitment

Potential participants at the Kellogg and PM&R clinics were informed of the study by clinic staff members during check in. If interested, participants were given a paper survey to complete. Completed surveys were returned to clinic staff members. In addition, participants were recruited at the monthly low-vision support group offered at the Kellogg Eye Center. At the family medicine clinic, study staff members were posted in the lobby and informed potential participants of the study. Those interested in participating were handed a survey, which was returned to study staff at completion. Recruitment occurred over 19 months from September 2019 to March 2021, with recruitment delays experienced due to the COVID-19 pandemic.

### Procedures

At each of the 3 clinic sites, an information sheet explaining the purpose of the survey, as well as a self-administered paper copy of the survey, were given to potential participants by clinic or study staff members. Participants then had the option to fill out the survey at any point during their clinic visit and return the paper survey to the clinic or study staff members upon completion. The assessment survey was self-administered by the respondent; however, in cases where assistance was needed for survey completion, participants could select a friend or caregiver accompanying them for assistance. In the case of participants recruited from the low vision clinic, clinic staff members were available to help respondents complete the survey.

### Survey Instrument

The study survey used was a 46-item investigator-developed paper-based instrument that surveyed participants about their knowledge, use, and perceptions of DVAs, their interest to participate in related future studies, and demographic questions (Multimedia Appendix 1). Questions regarding knowledge and use of DVAs were presented in 6 closed, multiple-choice format questions with some including an “other” option for further elaboration. There was one additional open-ended question that asked participants to give examples of potential use cases of DVAs that aligned with their interests. Participants were also asked to rate their concerns about 5 specific issues related to the use of DVAs, and one opportunity to voice their own concern. These ratings used a 5-point Likert scale (0=not concerned at all; 4=highly concerned) and were used to measure concerns relating to privacy, security, confidentiality, accuracy, and reliability of the DVAs. The questions about privacy, security, and confidentiality included definitions of the respective terms to clearly define the specific concern. “Privacy” referred to freedom to opt out of monitoring and to determine how personal data is used. “Security” referred to protection of personal data from loss or theft. “Confidentiality” was defined as the obligation of trusted persons to keep data safe and held in confidence. To assess participants’ interest in participating in future studies relating to DVAs, 3 closed multiple choice questions were asked.

The next set of questions presented assessed whether participants had a visual disability. If participants endorsed having a visual disability, 9 additional questions were presented asking about the nature of their disability, access to care, and access to resources. These questions were presented in mixed format. Participants who did not have visual disabilities were asked to skip to the next section, which was focused on physical disability.

The physical disability portion of the survey asked questions generically about disability, which allowed participants to self-identify and describe the nature of their disability. Those endorsing no physical disability were asked to skip to the final set of questions on demographics; whereas those who did endorse a physical disability were asked mixed format questions about the nature of their disability, access to care, and access to resources. As participants were allowed to self-identify with having a physical disability, this study included a wide range of physical disabilities and documented disability within the electronic health record was not required by any participants; rather, participants were asked to endorse whether they had any kind of physical disability and if so, questions probed into the nature of their self-reported disability.

Given that participants with visual disabilities were expected to have more difficulty taking the survey and would require assistance, a shortened 34-item survey presented in large print was administered to participants recruited from the Kellogg low vision clinic (Multimedia Appendix 2). The 34-item survey contained the same questions as the aforementioned 46-item instrument, except it excluded the question set focused on physical disabilities. Both versions of the survey were designed to be completely anonymous, with no protected health information or HIPAA (Health Insurance Portability and Accountability Act)-protected data collected. See MultimediaAppendices 1  2 for the survey instrument.

Participants gave their completed surveys to study or clinic staff members, who then placed the surveys into a box clearly labeled with the study title. After the collection of the completed surveys, the data were entered into a secure web application REDCap (Research Electronic Data Capture; Vanderbilt University) by approved study personnel. The entered data were verified through a 10% random sampling for entry errors. Incomplete survey responses were also retained and collected during this study.

### Statistical Analysis

The survey data were analyzed using descriptive statistics including frequencies, means, and SD. To determine differences in responses between subgroups, bivariate associations between subgroups and demographics, DVA use, and DVA concerns were performed using chi-square tests or Fisher exact tests as appropriate depending on the numbers in each group. Differences were examined by previous DVA use, clinic site, visual disability status, physical disability status, and willingness to participate in future research studies. All analyses were performed using SAS Software (version 9.4; SAS Institute Inc).

### Ethical Considerations

Our study was registered under a self-exemption process at the University of Michigan, classified under EXEMPTION 2 (i) and 2 (ii) as described in 45 CFR 46.104(d)(IRBMED HUM00156811). Participants were handed an information sheet outlining the risks and benefits of the study before completing the survey. All study materials were stored in locked file cabinets in the locked office of the principal investigator. All data were collected anonymously and reported in the aggregate, and participant responses cannot be linked to individual participants. No financial or nonfinancial incentives were offered for study participation.

## Results

### Demographics

A total of 218 eligible participants completed the survey. The majority of the sample was White (71.6%, 156/218) and female (61.7%, 124/201) with an average age of 52.6 (SD 18.9) years. The education levels of the participants varied, with the largest portion having a graduate degree (35.0%, 71/203), followed by a split between those who have completed a bachelor’s degree (20.2%, 41/203) and those who have completed some college education (19.7%, 40/203). Participants’ employment status was primarily full-time (40.8%, 82/201) followed by retired (29.9%, 60/201). Roughly half of the participant population earned an annual income of US $75,000 or higher (46.3%, 88/190) and was insured through their employer (54.1%, 118/218). Almost half of the participant population (49.5%, 101/204) indicated interest in participating in an additional research study that provides DVAs for use (Table 1).

**Table 1.** Demographics of all study participants.

Variable	Values
Age (years; n=217), mean (SD)	52.6 (18.9)
Site (n=218), n (%)	
Physical Medicine and Rehabilitation	84 (38.5)
Briarwood Family Medicine	80 (36.7)
Kellogg Eye Center	54 (24.8)
Gender (n=201), n (%)	
Male	76 (37.8)
Female	124 (61.7)
Other	1 (0.5)
Race^a^ (n=218), n (%)	
White	156 (71.6)
Black or African American	34 (15.6)
Asian	10 (4.6)
American Indian or Alaska Native	3 (1.4)
Native Hawaiian or other Pacific Islander	0 (0)
Other	8 (3.7)
Not reported	13 (6.0)
Hispanic, Latino, or Spanish origin (n=199), n (%)	
Yes	14 (7.0)
No	185 (93.0)
Living situation^a^ (n=218), n (%)	
Live alone	43 (19.7)
With spouse or other companion	121 (55.5)
With adult children	24 (11.0)
With young children	28 (12.8)
With siblings, parents, or other guardian	21 (9.6)
Other	11 (5.0)
Not reported	12 (5.5)
Income (n=190), n (%)	
Under US $25,000	31 (16.3)
US $25,000 - US $49,000	23 (12.1)
US $50,000 - US $74,999	33 (17.4)
US $75,000 or higher	88 (46.3)
Do not know	15 (7.9)
Insurance^a^ (n=218), n (%)	
Insured through an employer	118 (54.1)
Insurance purchased directly from company	18 (8.3)
Medicare	71 (32.6)
Medicaid	25 (11.5)
No insurance	2 (0.9)
Other	11 (5.0)
Not reported	16 (7.3)
Education (n=203), n (%)	
High school diploma or less	33 (16.3)
Some college	40 (19.7)
Associate’s degree, trade school or apprenticeship	18 (8.9)
Bachelor’s degree	41 (20.2)
Graduate degree	71 (35.0)
Employment status (n=201), n (%)	
Employed part-time	17 (8.5)
Employed full-time	82 (40.8)
Retired	60 (29.9)
Currently on disability	26 (12.9)
Laid off or unemployed	16 (8.0)
General health (n=206), n (%)	
Poor	11 (5.3)
Fair	40 (19.4)
Good	98 (47.6)
Very good	50 (24.3)
Excellent	7 (3.4)
Previously Used DVA^b^ (n=218), n (%)	
Yes	152 (69.7)
No	66 (30.3)
Willing to use DVA in the future (n=212), n (%)	
Yes	131 (61.8)
Maybe	47 (22.2)
No	34 (16.0)
Has any vision-related disabilities (n=211), n (%)	
Yes	77 (36.5)
No	134 (63.5)
Has any physical disabilities^c^ (n=156), n (%)	
Yes	47 (30.1)
No	109 (69.9)

a Participants could select multiple categories in these questions resulting in percentages that may add to more than 100%.

b DVA: digital voice assistant.

c Questions about physical difficulties were not asked at the Kellogg site. Number missing reflects this.

### General Use of DVAs

The results of the survey showed that DVAs are being used for various tasks and there is a willingness to use them in the future. The majority of all participants had previously used a DVA (69.7%, 152/218) and were or might be willing to use DVAs in the future (84.0%, 178/212) including 57.1% (36/63) of participants who had never used a DVA before (Table 1). Among the 152 participants who had used a DVA, Siri was the most highly used DVA (67.8%, 103/152). Other commonly used DVAs were Alexa and Google Assistant which were both used similarly by about nearly half (70/152 and 71/152, respectively) of participants. Of those who did report DVA use, the majority accessed the tool from their personal cell phone (88.8%, 135/152), and the main task was to answer questions (75.0% 114/152), with entertainment (57.2%, 87/152), accessing information online (55.9%, 85/152), and setting timers (51.3%, 78/152) also being frequent tasks done with a DVA. While many of the participants have used DVAs, the frequency of this use was split between those who use it rarely or sometimes (57.4%, 85/148) and those who use it often or always (42.6%, 63/148). Although the participants use DVAs, the majority did not rely on the DVA for everyday needs (57.0%, 85/149; Table 2).

**Table 2.** Digital voice assistant use characteristics among those who had previously used a digital voice assistant.

Variable	Participants, n (%)
DVA^a^ use (n=152)	
Alexa or Amazon Echo	70 (46.1)
Cortana	17 (11.2)
Siri	103 (67.8)
Google assistant	71 (46.7)
Google now	15 (9.9)
Other	6 (3.9)
None selected	0 (0)
DVA device (n=152)	
Personal cell phone	135 (88.8)
Personal Google home or Google Home Mini	23 (15.1)
Personal Echo or Echo Dot	43 (28.3)
Personal iPad or iPod	40 (26.3)
Personal tablet, laptop, or desktop computer	47 (30.9)
Device owned by another person	16 (10.5)
Other	9 (5.9)
None selected	2 (1.3)
DVA^a^ tasks (n=152)	
Answering questions	114 (75.0)
Managing schedules or calendars	61 (40.1)
Setting timers	78 (51.3)
Accessing information online	85 (55.9)
Entertainment	87 (57.2)
Other	25 (16.4)
None selected	1 (0.7)
DVA use frequency (n=148)	
Rarely	47 (31.8)
Sometimes	38 (25.7)
Often	40 (27.0)
Always	23 (15.5)
Rely on DVA for everyday needs, (n=149)	
Yes	41 (27.5)
Sometimes	23 (15.4)
No	85 (57.0)

a DVA: digital voice assistant; participants could select multiple categories in these questions resulting in percentages that may add to more than 100%.

### DVA Use and Visual Disabilities

Among respondents, 36.5% (77/211) reported having visual disabilities. Of the participants who reported having any visual disability, 81.6% (62/76) found it mildly to extremely difficult to perform activities because of their vision impairment and 41.9% (31/74) reported having vision-related needs not being met. Only 38.2% (26/68) of participants with visual disabilities indicated being very or highly satisfied with current services and devices, revealing a crucial need for improvement.

A greater proportion of participants with visual disabilities reported having never used DVA (39.0%, 30/77) than those without visual disabilities (24.6%, 33/134; *P*=.03). Most participants with visual disabilities who had used a DVA did so on their personal cell phone (53.2%, 41/77) but this was significantly less than the proportion of those without visual disabilities who use DVAs on cell phones (68.7%, 92/134) (*P*=.03). As in the entire sample, the most common DVA task was answering questions regardless of visual disability status, but this task was done by fewer people with visual disabilities (40.3%, 31/77) than those without visual disabilities (60.4%, 81/134; *P*=.005). Those with visual disabilities indicated relying on DVA sometimes or always (63.8%, 30/47), more often than those without visual disabilities (32.7%, 33/101; *P*=.001). In addition, participants with visual disabilities were equally as willing to potentially use DVAs in the future (85.3%, 64/75) as those without visual disabilities (85.0%, 113/133; *P*=.78). Within the group of participants who had never used a DVA, those with and those without visual disabilities had similar willingness to potentially use DVAs in the future (62.1%, 18/29 vs 56.3%, 18/32, respectively; *P*=.64). Those with visual disabilities were also more willing to grant access to past data for DVA studies than those without visual disabilities (22.5%, 16/71 vs 13.1%, 17/130; *P*=.01; Table 3).

**Table 3.** Digital voice assistant use characteristics by disability status. *P* values are derived from chi-square tests unless otherwise noted.

	Visual disabilities			Physical disabilities		
	No (N=134), n (%)	Yes (N=77), n (%)	*P* value	No (N=109), n (%)	Yes (N=47), n (%)	*P* value
DVA^a^ use						
Alexa or Amazon Echo	49 (36.6)	21 (27)	.17	46 (42.2)	10 (21)	.01
Cortana	12 (9.0)	5 (6)	.53	9 (8.3)	5 (11)	.76^b^
Siri	72 (53.7)	28 (36)	.02	63 (57.8)	20 (43)	.08
Google assistant	48 (35.8)	21 (27)	.20	39 (35.8)	17 (36)	.96
Google now	10 (7.5)	5 (6)	.79	12 (11.0)	1 (2)	.11^b^
Never used DVA	33 (24.6)	30 (39)	.03	23 (21.1)	16 (34)	.09
Other	1 (0.7)	5 (6)	.03^b^	1 (0.9)	0 (0)	.99^b^
None selected	0 (0)	1 (1)	.36^b^	0 (0)	0 (0)	N/A^c^
DVA device						
Personal cell phone	92 (68.7)	41 (53)	.03	80 (73.4)	27 (57)	.049
Personal Google home or Google home mini	15 (11.2)	8 (10)	.86	14 (12.8)	4 (9)	.44
Personal Echo or Echo Dot	31 (23.1)	12 (16)	.19	26 (23.9)	6 (13)	.12
Personal iPad or iPod	23 (17.2)	15 (19)	.67	23 (21.1)	6 (13)	.22
Personal tablet, laptop, or desktop computer	26 (19.4)	21 (27)	.19	21 (19.3)	7 (15)	.51
Device owned by another person	11 (8.2)	5 (6)	.65	9 (8.3)	3 (6)	.99^b^
Other	4 (3.0)	5 (6)	.29^b^	5 (4.6)	0 (0)	.32^b^
None selected	33 (24.6)	30 (39)	.03	23 (21.1)	16 (34)	.09
DVA tasks						
Answering questions	81 (60.4)	31 (40)	.005	68 (62.4)	28 (60)	.74
Managing schedules or calendars	41 (30.6)	19 (25)	.36	38 (34.9)	11 (23)	.16
Setting timers	54 (40.3)	23 (30)	.13	50 (45.9)	14 (30)	.06
Accessing information online	54 (40.3)	30 (39)	.85	47 (43.1)	20 (43)	.95
Entertainment	61 (45.5)	25 (32)	.06	54 (49.5)	19 (40)	.30
Other	13 (9.7)	12 (16)	.20	12 (11.0)	2 (4)	.23^b^
None selected	0 (0)	1 (1)	.37^b^	0 (0)	0 (0)	N/A^c^
DVA use frequency			.02			.30
Rarely or sometimes	64 (64.0)	20 (43)		53 (62.4)	16 (52)	
Often or always	36 (36.0)	26 (57)		32 (37.6)	15 (48)	
Not reported^d^	34 (25.4)	31 (40)	24 (22.0)	16 (34)
Rely on DVA for everyday needs			.001			.08
Yes	22 (21.8)	18 (38)		17 (19.8)	12 (39)	
Sometimes	11 (10.9)	12 (26)		13 (15.1)	2 (6)	
No	68 (67.3)	17 (36)		56 (65.1)	17 (55)	
Not reported^d^	33 (24.6)	30 (39)	23 (21.1)	16 (34)
Willing to use DVA in future			.78			.82
Yes	81 (60.9)	49 (65)		70 (64.2)	27 (60)	
Maybe	32 (24.1)	15 (20)		24 (22.0)	12 (27)	
No	20 (15.0)	11 (15)		15 (13.8)	6 (13)	
Not reported^d^	1 (0.7)	2 (3)	0 (0)	2 (4)
Willing to share existing DVA data			.01			.36
Yes	17 (13.1)	16 (23)		15 (14.2)	7 (15)	
Maybe	25 (19.2)	9 (13)		21 (19.8)	7 (15)	
No	55 (42.3)	17 (24)		47 (44.3)	16 (35)	
Do not have a DVA	33 (25.4)	29 (41)		23 (21.7)	16 (35)	
Not reported^d^	4 (3.0)	6 (8)	3 (2.8)	1 (2)

a DVA: digital voice assistant participants; could select multiple categories in these questions resulting in percentages that may add to more than 100%. *P* values for these variables are testing the difference in proportion selecting “Yes” in an individual category by disability status.

b *P* value from the Fisher exact test.

c N/A: not applicable.

d These values were excluded from the chi-square tests, as well as the % calculations.

### DVA Use and Physical Disabilities

Among respondents, 47 (30.1%, 47/156) reported having a physical disability. Those with physical disabilities were more likely to report also having visual disabilities (29.8%, 14/47) than those who did not report having any physical disabilities (9.2%, 10/109, respectively; *P*=.001). Among the participants who possessed physical disabilities, 63.0% (29/46) found it moderately, very, or extremely difficult to perform everyday activities. The most common activities affected by the physical disability were walking or climbing stairs (87.2%, 41/47) and holding objects in one’s hands (31.9%, 15/47). Most participants (80.4%, 37/46) reported that their physical needs are being met. Still, only 55.6% (10/18) of participants with physical disabilities report being very or highly satisfied with the services or devices meeting their needs.

Participants with physical disabilities reported having never used DVA (34.0%, 16/47) more often than those without a physical disability (21.1%, 23/109), although the difference is not statistically significant (*P*=.09). The proportion of participants that used DVAs for certain tasks tended to be lower for those with physical disabilities than those without physical disabilities but again these differences were not statistically significant. There was a statistically significant difference (*P*=.01) in the use of Alexa or Amazon Echo for those with physical disabilities vs those without physical disabilities (21.3%, 10/47 vs 42.2%, 46/109, respectively). Similarly to those with visual disabilities, the most common task participants with physical disabilities used DVAs for was answering questions (59.6%, 28/47). There was no significant difference in the participants’ reliance on DVA for everyday needs by physical disability status. Most participants with physical disabilities were willing, or possibly willing, to use DVA in the future (86.7%, 39/45). There is a significant difference of reliance on DVA with those without either disability being less reliant and those with visual or both visual and physical disabilities being more reliant (*P*=.01; Table 3).

### Concerns With DVAs

Overall, a large proportion of participants had at least a slight concern about privacy (86.2%, 175/203), security (89.1%, 180/202), confidentiality (86.7% 176/203), accuracy of information (81.7%, 165/202), and reliability of information (85.1%, 171/201). More specifically, roughly half of the participants had moderate to high concerns about the privacy (47.8%, 97/203), security (54.5%, 110/202), and confidentiality (51.7%, 105/203) of DVAs. The degree of concern about privacy, security, and confidentiality had no significant differences between the participants with and those without visual disabilities. We found a significant difference between concerns about accuracy (*P*=.04) and reliability (*P*=.048) of information from DVAs based on visual disability status with participants with visual disabilities tending to be moderately or highly concerned more often. The degree of concern about privacy, security, confidentiality, accuracy, and reliability had no significant differences between the participants with and those without physical disabilities. Despite these concerns, we generally did not see a significant decrease in DVA use, frequency, or reliance for those with higher levels of concern. The one exception was in those with high levels of concern about security who tended to use DVAs less frequently than those with slight or no concern about security (*P*=.04; Table 4).

**Table 4.** Perceptions of digital voice assistants overall and by disability statuses. *P* values are derived from chi-square tests.

Concerns	Overall (N=218), n (%)	Visual disabilities			Physical disabilities^a^		
		No (N=134), n (%)	Yes (N=77), n (%)	*P* value	No (N=109), n (%)	Yes (N=47), n (%)	*P* value
Privacy				.24			.71
Not concerned at all	28 (13.8)	14 (10.8)	14 (19)		9 (8.5)	4 (9)	
Slightly or somewhat concerned	78 (38.4)	51 (39.2)	27 (37)		42 (39.6)	15 (33)	
Moderately or highly concerned	97 (47.8)	65 (50.0)	32 (44)		55 (51.9)	27 (59)	
Not reported^b^	15 (6.9)	4 (3.0)	4 (5)	3 (2.8)	1 (2)
Security				.22			.72
Not concerned at all	22 (10.9)	11 (8.5)	11 (15)		6 (5.7)	4 (9)	
Slightly or somewhat concerned	70 (34.7)	49 (38.0)	21 (29)		37 (35.2)	14 (30)	
Moderately or highly concerned	110 (54.5)	69 (53.5)	41 (56)		62 (59.0)	28 (61)	
Not reported^b^	16 (7.3)	5 (3.7)	4 (5)	4 (3.7)	1 (2)
Confidentiality				.29			.54
Not concerned at all	27 (13.3)	14 (10.8)	13 (18)		8 (7.5)	6 (13)	
Slightly or somewhat concerned	71 (35.0)	49 (37.7)	22 (30)		37 (34.9)	14 (30)	
Moderately or highly concerned	105 (51.7)	67 (51.5)	38 (52)		61 (57.5)	26 (57)	
Not reported^b^	15 (6.9)	4 (3.0)	4 (5)	3 (2.8)	1 (2)
Accuracy of information				.04			.21
Not concerned at all	37 (18.3)	21 (16.2)	16 (22)		14 (13.2)	9 (20)	
Slightly or somewhat concerned	83 (41.1)	62 (47.7)	21 (29)		53 (50.0)	16 (35)	
Moderately or highly concerned	82 (40.6)	47 (36.2)	35 (49)		39 (36.8)	21 (46)	
Not reported^b^	16 (7.3)	4 (3.0)	5 (6)	3 (2.8)	1 (2)
Reliability of information				.048			.13
Not concerned at all	30 (14.9)	16 (12.4)	14 (19)		9 (8.5)	9 (20)	
Slightly or somewhat concerned	81 (40.3)	60 (46.5)	21 (29)		49 (46.2)	17 (38)	
Moderately or highly concerned	90 (44.8)	53 (41.1)	37 (51)		48 (45.3)	19 (42)	
Not reported^b^	17 (7.8)	5 (3.7)	5 (6)	3 (2.8)	2 (4)

a Participants recruited from Kellogg Eye Center were not asked questions pertaining to physical disabilities, so the total number of people reporting physical disability status is a subset of the total sample.

b These values were excluded from the chi-square tests, as well as the % calculations.

## Discussion

### Principal Findings

In this study, we found the majority of participants had previous experience with DVAs and that there was interest in using them in the future. Looking specifically at those with self-reported disabilities, many adults with physical disabilities and visual disabilities do not regularly use DVAs, however, those with visual disabilities have a greater reliance on the technology. Overall, participants were moderately concerned with the issues of privacy, security, confidentiality, accuracy, and reliability of DVAs. Roughly half of those with visual disabilities had moderate or high concern for privacy, security, and confidentiality, but these numbers do not drastically differ from those without visual disabilities, or with or without physical disabilities, nor does it seem to affect their use and reliance on the technology. Previous literature has discussed smartphone-based low vision aids where DVAs are briefly examined among other smartphone tools for low vision usability [17,18]. The use of DVAs for people with disabilities, especially for the purposes of living independently, is a rapidly expanding field of study [15]. Many recent studies have focused on the accessibility, perceptions, and feasibility of the applications of DVAs, but few have evaluated the actual use cases and frequency of DVA use among individuals with visual or physical disabilities. This is an important step toward understanding and developing technology that better meets the needs of these individuals to improve health outcomes and quality of life.

### Regularity of DVA Use

Overall, it was observed that more than half of the participants used DVAs only “sometimes” or “rarely.” Despite the lack of current use when the survey instrument was completed, more than half of the participants were willing to use DVAs in the future. This was perhaps a function of age, seeing that the average age of participants was older at 52.6 years old. From the survey, it was observed that the dominant use of DVAs was found in smartphones. Smartphone use among older age groups differs from those younger as a 2024 study from the Pew Research Center reported that 91% of adults aged between 50‐64 years and 79% of adults aged 65 years and older own smartphones in the United States, compared with 99% of adults aged 18‐29 years [1]. Using smart speakers as DVAs in populations of older adults in low socioeconomic positions has also proved to be promising, though more research should be conducted to promote the answering of health-related questions [19]. The additional smartphone functions and tools available for consumers are not necessarily used by older populations. While lower DVA use is more common among older adults, this could be related to a number of factors including lesser comfort with the technology, lower digital literacy, usability factors, technology learning curves, and other personal hesitations.

### Perceptions of DVAs

The perceptions of DVAs were quite similar across those with and those without physical and visual disabilities. Overall, about half of participants expressed a moderate to high concern with the security and reliability of DVAs. It was interesting to note that those with visual disabilities were less concerned with privacy and confidentiality issues than those without visual disabilities. Concerns regarding online data privacy, security, and reliability are not novel, as literature spanning decades has documented these concerns. With specific regards to data privacy, DVAs represent an interesting use case as large data giants such as Facebook (Meta) and Amazon are increasing their use of personal user data for advertising, as well as individual targeting and tailoring of products and services. DVA companies have a reputation for lacking transparency with users about privacy protection which can lead to a lack of trust among users [20]. Many DVAs rely on the “activation keyword approach,” where devices listen for a specific keyword before they start recording, which means that these technologies are always listening, unless muted. It also means that DVAs are susceptible to false activation, which means that recording could be unintentionally turned on at a time when users are not aware. Overall, there needs to be improved transparency and additional user controls for personal recording for DVAs and their devices. However, despite the concern over data privacy, 23% of participants reporting visual disabilities and 15% of participants reporting physical disabilities in our study were willing to grant access to their past existing data or share future data for future DVA studies.

The landscape of DVAs along with the integration of generative artificial intelligence (AI) is everchanging, which could lead to evolving data privacy and data quality concerns regarding DVAs. Since the release of ChatGPT by OpenAI in 2022, the interest and number of publications regarding “trust” in conversational agents has risen rapidly. Before 2024, computer science held the vast majority of publications regarding trust in conversational agents; however, the field of medicine has shown greater growth in interdisciplinary research with conversational agents over the last 8 years [21]. Applications of AI in health systems will require close monitoring and transparency for patients to feel comfortable granting access [22]. Recent research has shown that many individuals feel relatively comfortable sharing their data with national health organizations and universities, but much less willing to share with private commercial organizations such as insurance and technological companies [23]. This further emphasizes the importance of exploring utility and applications of DVAs and AI in health care and research contexts.

### Reliance on DVAs by Those With Visual Disabilities

The survey showed that those with visual disabilities had a greater reliance on DVAs than those without visual disabilities. This is likely due to those with visual disabilities relying on voice commands to read and assist them with tasks they otherwise would not be able to do. In terms of specific activities, participants with visual disabilities reported using DVAs more frequently for answering questions than those without visual disabilities.

### DVA Use by Those With Physical Disabilities

The survey showed that the DVA use cases of those with physical disabilities did not differ significantly from those without physical disabilities. Due to the nature of the relatively small convenience sample, there was not a large degree of heterogeneity in terms of types of physical disabilities. Those with physical disabilities were shown to rely more on DVAs than those without. The finding of increased reliance is supported by previous interview-based research that noted DVAs promote autonomy by increasing the ability to perform daily activities and tasks that were normally obstructed by mobility or other barriers [24].

### Strengths and Limitations

To our knowledge, this is one of the first studies to assess the utility and perceptions of DVAs for adults with specific focus on those with visual and physical disabilities. Despite the novelty of the study, there are multiple limitations to consider that can guide future exploration. It is crucial to note that due to the COVID-19 pandemic, data collection was disrupted with delays in analysis. Despite the lag experienced, the study begins to fill a literature gap on the use of DVAs, particularly from the perspective of those living with visual and physical disabilities.

This study is not without limitations. The study findings relied on participant self-reported information, which may introduce response or recall bias, such as participants not remembering, or choosing not to share information related to disabilities or DVA use. Moreover, this study used a convenience sample, which is a limitation to our approach. In addition, participants were recruited from one health system, so results may not be generalizable to other populations. To reduce participant burden among those with visual disabilities, the survey was shortened. This may have overlooked potential dual disability concerns (physical disabilities among those with visual disabilities). In addition, the study survey did not capture the specifics related to the reported disability, such as etiology, onset, severity and overall quality of life. In addition, the survey instrument used was created specifically for this study and not validated. Despite these limitations, our study was intended to be exploratory in nature and serve as a foundation for future work in this field.

### Future Perspectives

Future work should examine DVA use through device use data recordings and compare this across different disabilities and functional levels. Additional areas of investigation should incorporate qualitative approaches to more fully elucidate the ways that DVAs have been used, or could be used, to support the health of older adults and those individuals living with disabilities. Not only does this have great implications for the more than 25% of individuals in the United States living with a disability [25], but it also has great implications for the number of aging individuals who may benefit from DVA use (eg, social isolation and improved independence).

### Conclusions

Self-reported use of DVA varied across the surveyed participants. Tasks such as answering questions and seeking more information were the most used functions of DVAs by individuals with physical and visual disabilities. Participant perceptions of DVAs, regardless of disability status, suggest that concerns with data privacy, security, confidentiality, reliability, and accuracy exist.

## Supplementary material

10.2196/66185 Multimedia Appendix 1 Digital personal assistant pilot study.

10.2196/66185 Multimedia Appendix 2 Digital personal assistant pilot study - large print.

## Abbreviations

AI: artificial intelligence
DVA: digital voice assistant
HIPAA: Health Insurance Portability and Accountability Act
PM&R: physical medicine and rehabilitation
REDCap: Research Electronic Data Capture
