# Supplementary material for: Exploring the Utility of Digital Voice Assistants for Primary Care Patients, Including Those With Physical and Visual Disabilities: Cross-Sectional Study
Source: JMIR Mhealth Uhealth. 2025 Aug 14;13:e66185. doi: 10.2196/66185 (PMC12352795; doi:10.2196/66185)
Supplement: Multimedia Appendix 1 [file mhealth-v13-e66185-s001.docx]

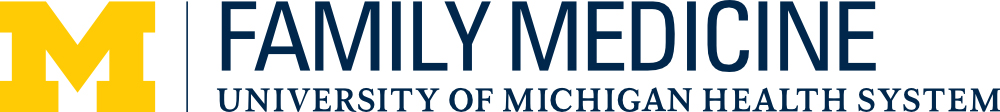


**Study Title:** **Digital Personal Assistant Pilot Study**

1. **I have read and understand the information sheet provided with this survey, and I agree to provide my responses to the study team:**
   1. Yes
   2. No ***[please hand this back to the clinic staff or discard]***
2. How old are you? _____________ years **If you are under 18 years old, please do not take this survey.**
3. Are you answering for yourself or for another person?
4. Self
5. Another *[be aware that all questions below are for the person you are answering for]*

## *This section asks you questions about your knowledge, use, and perceptions of digital personal assistants*

1. Have you ever used any of the following digital personal assistants, (i.e., device or software that respond to and use voice-commands to help answer questions and complete simple tasks)? *(Circle all that apply)*
2. Alexa or Amazon Echo
3. Cortana
4. Siri
5. Google Assistant
6. Google now
7. I have never used a digital personal assistant **[if you answered F, skip to Question 9]**
8. Other, please describe_____________________________________________________
9. On what device have you used a digital personal assistant? *(Circle all that apply)*
10. Personal cell phone
11. Personal Google Home or Google Home Mini
12. Personal Echo or Echo Dot
13. Personal iPad / iPod
14. Personal Tablet / laptop / desktop computer
15. A device owned by another person
16. Other, please describe_____________________________________________________
17. What kinds of tasks have you used a digital personal assistant for? *(Circle all that apply)*
18. Answering questions
19. Managing schedules / Calendar
20. Setting timers
21. Accessing information online
22. Entertainment
23. Other, please describe_______________________________________________________________
24. Approximately how often do you use a digital personal assistant each day?
25. Rarely
26. Sometimes
27. Often
28. Always
29. Do you rely on personal digital assistants to help meet your everyday needs?
30. Yes
31. No
32. Sometimes
33. Would you be willing to use a digital personal assistant in the future?
34. Yes
35. No, explain why: __________________________________________________________________
36. Maybe
37. If you had access to a digital personal assistant, what kinds of things would you be interested in using it for in the future? *(write out the top three things below)*:

1. __________________________________________________________________________________

2. __________________________________________________________________________________

3. __________________________________________________________________________________

1. Please rate how concerned you are about the following issues in relation to a digital personal assistant?

|  | Not concerned at all | Slightly concerned | Somewhat concerned | Moderately concerned | Highly concerned |
| --- | --- | --- | --- | --- | --- |
| - 1. Privacy (referring to your individual right to be free from monitoring without permission, and to decide how your personal data is used) | 📺 | 📺 | 📺 | 📺 | 📺 |
| - 1. Security (referring to the protection of your private data from accidental loss or theft) | 📺 | 📺 | 📺 | 📺 | 📺 |
| - 1. Confidentiality (referring to the obligation that people who have been trusted to have access to your personal data have to keep that data safe and hold it in confidence) | 📺 | 📺 | 📺 | 📺 | 📺 |
| - 1. Accuracy of information | 📺 | 📺 | 📺 | 📺 | 📺 |
| - 1. Reliability of information | 📺 | 📺 | 📺 | 📺 | 📺 |
| - 1. Other, please describe:   _____________________________________ | 📺 | 📺 | 📺 | 📺 | 📺 |

***Our research team is exploring the development of a research study looking at how people might use digital personal assistants to help with everyday activities or to manage their health conditions. In this next section we want to ask you about your interest in participating in any future research studies focusing on digital personal assistants.***

1. Would you be interested in participating in a research study that provides digital personal assistants for use?
2. Yes
3. No
4. Would you be willing to share any data that may be collected by that device for research purposes?
5. Yes
6. No
7. Maybe
8. If you currently have a personal digital assistant, would you be willing to share the data that the device has already collected?
9. Yes
10. No
11. Maybe
12. I do not have a personal digital assistant

***This next set of questions focuses on vision-related disabilities.***

1. Do you have any vision-related disabilities?
   1. Yes
   2. No **(If you answered No, skip to Question #25)**
2. How would you describe your vision-related disability? *(Please fill in the blank below)*

_________________________________________________________________________________________

1. What is the cause of your vision-related disability? *(Please fill in the blank below)*

_________________________________________________________________________________________

1. Because of your vision-related disability, how difficult is it for you to perform everyday activities? (i.e. managing finances, housekeeping, using the telephone, watching TV)
2. Not difficult at all
3. Mildly difficult
4. Moderately difficult
5. Very difficult
6. Extremely difficult
7. Have you delayed getting needed healthcare due to your vision-related disability?
   1. Yes, please describe ________________________________________________________________
   2. No
8. Do you have vision-related needs that are not being met?
   1. Yes, please describe ________________________________________________________________
   2. No
9. What services or equipment/devices have you received for your vision-related disability? *(Please fill in the blank below)*

_________________________________________________________________________________________

1. How satisfied are you that your current services or assistive devices meet your needs?
   1. Not at all satisfied
   2. Slightly satisfied
   3. Somewhat satisfied
   4. Very satisfied
   5. Highly satisfied
2. Was there significant delay between being diagnosed with your vision disability (e.g. vision loss) and when you received services or assistive equipment (e.g., education supports, technology, devices etc.) related to your disability?
3. Yes
4. No **(If you answered No, skip to Question #25)**
5. Don’t know
6. Approximately how long was that delay? Please record length of time in months and/or years:

__________ months __________ years

***This set of questions focuses on physical disabilities.***

1. Do you have any physical disabilities?
   1. Yes
   2. No **(If you answered No, skip to Question #37)**
2. What kind of physical disability do you have? *(Circle all that apply)*
3. Difficulty walking or climbing stairs
4. Difficulty holding or using objects in your hands
5. Difficulty caring for yourself (e.g. bathing or dressing)
6. Difficulty living independently (e.g. difficulty doing errands alone such as visiting a doctor’s office or shopping)
7. Other, please describe_____________________________________________________
8. What is the cause of your physical disability? *(Please fill in the blank below)*
9. Because of your physical disability, how difficult is it for you to perform everyday activities (i.e. taking a bath, housekeeping)?
10. Not difficult at all
11. Mildly difficult
12. Moderately difficult
13. Very difficult
14. Extremely difficult
15. Do you use any of the following accessibility equipment? *(Circle all that apply)*
16. Support cane
17. Crutches
18. Walker
19. Wheelchair
20. Battery-operated scooter
21. Support rail
22. Other, please describe_______________________________________________________________
23. None
24. Do you have any mobility limitations? *(Circle all that apply)*
25. No
26. Yes, limited head/neck movement
27. Yes, limited arm movement
28. Yes, limited balance when seated
29. Yes, I have some other mobility limitation, please describe: *________________________________*
30. Have you delayed getting needed healthcare due to your physical disability?
31. No
32. Yes, please describe ________________________________________________________________
33. Do you have physical needs that are not being met?
    1. No
    2. Yes, please describe ________________________________________________________________
34. What services/assistive equipment have you received for your physical disability?
35. List all: ____­­­­­­­­_____________________________________________________________________
36. None **(If you answered No, skip to Question #37)**
37. How satisfied are you that your current services or assistive devices meet your needs?
    1. Not at all satisfied
    2. Slightly satisfied
    3. Somewhat satisfied
    4. Very satisfied
    5. Highly satisfied
38. Was there significant delay between being diagnosed with your physical disability (e.g. mobility limitations) and when you received services or assistive equipment (e.g., education supports, mobility tools, etc.) related to your disability?
39. Yes
40. No **(If you answered No, skip to Question #37)**
41. Don’t know
42. Approximately how long was that delay? Please record length of time in months and/or years:

__________ months __________ years

## *This final set of questions helps us to understand more about you.*

1. In general, how would you describe your health?
2. Poor
3. Fair
4. Good
5. Very good
6. Excellent
7. Which of the following best represents your racial or ethnic heritage? *(Circle all that apply)*
8. Caucasian/White
9. Black or African American
10. Asian
11. American Indian or Alaska Native
12. Native Hawaiian or other Pacific Islander
13. Other, please describe ______________________________________________________________
14. Are you Hispanic, Latino, or Spanish origin?
15. Yes
16. No
17. Don’t know
18. What best describes your current living situation? *(Circle all that apply)*
19. Live alone
20. With spouse or other companion
21. With adult children
22. With young children
23. With siblings/parents/or other guardian
24. Other, please describe ______________________________________________________________
25. Do you live in a/an:
26. House
27. Apt/Condo/Townhouse
28. Nursing home
29. Retirement Community
30. Independent living community
31. Other, please describe ______________________________________________________________
32. What is the combined annual household income?
33. Under $25,000
34. $25,000–$49,999
35. $50,000–$74,999
36. $75,000 or higher
37. Don’t know
38. What is your gender?
39. Male
40. Female
41. Other, please describe ______________________________________________________________
42. Which best describes the type of health insurance or health coverage plan you have? *(Circle all that apply)*
43. Insurance through a current or former employer
44. Insurance purchased directly from an insurance company
45. Medicare
46. Medicaid
47. No insurance coverage
48. Other, please describe ______________________________________________________________
49. What is the highest level of school you completed?
50. High school diploma or less
51. Some college
52. Associate’s degree / Trade school or apprenticeship
53. Bachelor's degree
54. Graduate degree
55. What is your current employment status?
56. Employed part-time
57. Employed full-time
58. Retired
59. Currently on disability
60. Laid off or unemployed

***This concludes the survey. Thank you so much for your time today!***
