# Supplementary material for: Exploring the Utility of Digital Voice Assistants for Primary Care Patients, Including Those With Physical and Visual Disabilities: Cross-Sectional Study
Source: JMIR Mhealth Uhealth. 2025 Aug 14;13:e66185. doi: 10.2196/66185 (PMC12352795; doi:10.2196/66185)
Supplement: Multimedia Appendix 2 [file mhealth-v13-e66185-s002.docx]

**
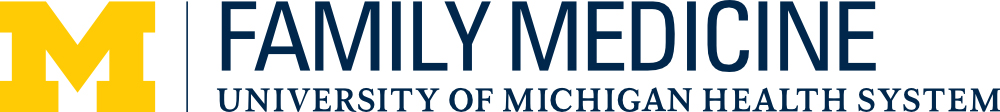
**

**Digital Personal Assistant Pilot Study**

1. **I have read and understand the information sheet provided with this survey, and I agree to provide my responses to the study team:**
   1. **Yes**
   2. **No [please hand this back to the clinic staff or discard]**
2. **How old are you? _____________ years If you are under 18 years old, please do not take this survey.**
3. **Are you answering for yourself or for another person?**
4. **Self**
5. **Another [be aware that all questions below are for the person you are answering for]**

## This section asks you questions about your knowledge, use, and perceptions of digital personal assistants

1. **Have you ever used any of the following digital personal assistants, (i.e., device or software that respond to and use voice-commands to help answer questions and complete simple tasks)? (Circle all that apply)**
2. **Alexa or Amazon Echo**
3. **Cortana**
4. **Siri**
5. **Google Assistant**
6. **Google now**
7. **I have never used a digital personal assistant [if you answered F, skip to Question 9]**
8. **Other, please describe___________________________________**
9. **On what device have you used a digital personal assistant? (Circle all that apply)**
10. **Personal cell phone**
11. **Personal Google Home or Google Home Mini**
12. **Personal Echo or Echo Dot**
13. **Personal iPad / iPod**
14. **Personal Tablet / laptop / desktop computer**
15. **A device owned by another person**
16. **Other, please describe__________________________**
17. **What kinds of tasks have you used a digital personal assistant for? (Circle all that apply)**
18. **Answering questions**
19. **Managing schedules / Calendar**
20. **Setting timers**
21. **Accessing information online**
22. **Entertainment**
23. **Other, please describe___________________________________**
24. **Approximately how often do you use a digital personal assistant each day?**
25. **Rarely**
26. **Sometimes**
27. **Often**
28. **Always**
29. **Do you rely on personal digital assistants to help meet your everyday needs?**
30. **Yes**
31. **No**
32. **Sometimes**
33. **Would you be willing to use a digital personal assistant in the future?**
34. **Yes**
35. **No, explain why: _______________________________________**
36. **Maybe**
37. **If you had access to a digital personal assistant, what kinds of things would you be interested in using it for in the future? (write out the top three things below):**

**1. ______________________________________________________**

**2. ______________________________________________________**

**3. ______________________________________________________**

1. **Please rate how concerned you are about the following issues in relation to a digital personal assistant?**

|  | **Not concerned at all** | **Slightly concerned** | **Somewhat concerned** | **Moderately concerned** | **Highly concerned** |
| --- | --- | --- | --- | --- | --- |
| - 1. **Privacy (referring to your individual right to be free from monitoring without permission, and to decide how your personal data is used)** | **📺** | **📺** | **📺** | **📺** | **📺** |
| - 1. **Security (referring to the protection of your private data from accidental loss or theft)** | **📺** | **📺** | **📺** | **📺** | **📺** |
| - 1. **Confidentiality (referring to the obligation that people who have been trusted to have access to your personal data have to keep that data safe and hold it in confidence)** | **📺** | **📺** | **📺** | **📺** | **📺** |
| - 1. **Accuracy of information** | **📺** | **📺** | **📺** | **📺** | **📺** |
| - 1. **Reliability of information** | **📺** | **📺** | **📺** | **📺** | **📺** |
| - 1. **Other, please describe:** | **📺** | **📺** | **📺** | **📺** | **📺** |

**Our research team is exploring the development of a research study looking at how people might use digital personal assistants to help with everyday activities or to manage their health conditions. In this next section, we want to ask you about your interest in participating in any future research studies focusing on digital personal assistants.**

1. **Would you be interested in participating in a research study that provides digital personal assistants for use?**
2. **Yes**
3. **No**
4. **Would you be willing to share any data that may be collected by that device for research purposes?**
5. **Yes**
6. **No**
7. **Maybe**
8. **If you currently have a personal digital assistant, would you be willing to share the data that the device has already collected?**
9. **Yes**
10. **No**
11. **Maybe**
12. **I do not have a personal digital assistant**

**This next set of questions focuses on vision-related disabilities.**

1. **Do you have any vision-related disabilities?**
   1. **Yes**
   2. **No (If you answered No, skip to Question #25)**
2. **How would you describe your vision-related disability? (Please fill in the blank below)**

**________________________________________________________**

1. **What is the cause of your vision-related disability? (Please fill in the blank below)**

**________________________________________________________**

1. **Because of your vision-related disability, how difficult is it for you to perform everyday activities? (i.e. managing finances, housekeeping, using the telephone, watching TV)**
2. **Not difficult at all**
3. **Mildly difficult**
4. **Moderately difficult**
5. **Very difficult**
6. **Extremely difficult**
7. **Have you delayed getting needed healthcare due to your vision-related disability?**
   1. **Yes, please describe __________________________________**
   2. **No**
8. **Do you have vision-related needs that are not being met?**
   1. **Yes, please describe _____________________________________**
   2. **No**
9. **What services or equipment/devices have you received for your vision-related disability? (Please fill in the blank below)**

**________________________________________________________**

1. **How satisfied are you that your current services or assistive devices meet your needs?**
   1. **Not at all satisfied**
   2. **Slightly satisfied**
   3. **Somewhat satisfied**
   4. **Very satisfied**
   5. **Highly satisfied**
2. **Was there significant delay between being diagnosed with your vision disability (e.g. vision loss) and when you received services or assistive equipment (e.g., education supports, technology, devices etc.) related to your disability?**
3. **Yes**
4. **No (If you answered No, skip to Question #25)**
5. **Don’t know**
6. **Approximately how long was that delay? Please record length of time in months and/or years:**

**__________ months __________ years**

## This final set of questions helps us to understand more about you.

1. **In general, how would you describe your health?**
2. **Poor**
3. **Fair**
4. **Good**
5. **Very good**
6. **Excellent**
7. **Which of the following best represents your racial or ethnic heritage? (Circle all that apply)**
8. **Caucasian/White**
9. **Black or African American**
10. **Asian**
11. **American Indian or Alaska Native**
12. **Native Hawaiian or other Pacific Islander**
13. **Other, please describe __________________________________**
14. **Are you Hispanic, Latino, or Spanish origin?**
15. **Yes**
16. **No**
17. **Don’t know**
18. **What best describes your current living situation? (Circle all that apply)**
19. **Live alone**
20. **With spouse or other companion**
21. **With adult children**
22. **With young children**
23. **With siblings/parents/or other guardian**
24. **Other, please describe __________________________________**
25. **Do you live in a/an:**
26. **House**
27. **Apt/Condo/Townhouse**
28. **Nursing home**
29. **Retirement Community**
30. **Independent living community**
31. **Other, please describe __________________________________**
32. **What is the combined annual household income?**
33. **Under $25,000**
34. **$25,000–$49,999**
35. **$50,000–$74,999**
36. **$75,000 or higher**
37. **Don’t know**
38. **What is your gender?**
39. **Male**
40. **Female**
41. **Other, please describe __________________________________**
42. **Which best describes the type of health insurance or health coverage plan you have? (Circle all that apply)**
43. **Insurance through a current or former employer**
44. **Insurance purchased directly from an insurance company**
45. **Medicare**
46. **Medicaid**
47. **No insurance coverage**
48. **Other, please describe __________________________________**
49. **What is the highest level of school you completed?**
50. **High school diploma or less**
51. **Some college**
52. **Associate’s degree / Trade school or apprenticeship**
53. **Bachelor's degree**
54. **Graduate degree**
55. **What is your current employment status?**
56. **Employed part-time**
57. **Employed full-time**
58. **Retired**
59. **Currently on disability**
60. **Laid off or unemployed**

**This concludes the survey. Thank you so much for your time today!**
